# Large-field-of-view optical elastography using digital image correlation for biological soft tissue investigation

**DOI:** 10.1117/1.JMI.4.1.014505

**Published:** 2017-03-16

**Authors:** Daniel Claus, Marijo Mlikota, Jonathan Geibel, Thomas Reichenbach, Giancarlo Pedrini, Johannes Mischinger, Siegfried Schmauder, Wolfgang Osten

**Affiliations:** a Universität Stuttgart, Institut für Technische Optik, Stuttgart, Germany; b Universität Stuttgart, Institut für Materialprüfung, Werkstoffkunde und Festigkeitslehre, Stuttgart, Germany; c Eberhard Karls Universität Tübingen, Klinik für Urologie, Universitätsklinikum Tübingen, Tübingen, Germany

**Keywords:** elastography, image correlation, displacement measurement, strain measurement, soft tissue FE modeling

## Abstract

In minimally invasive surgery the haptic feedback, which represents an important tool for the localization of abnormalities, is no longer available. Elastography is an imaging technique that results in quantitative elastic parameters. It can hence be used to replace the lost sense of touch, as to enable tissue localization and discrimination. Digital image correlation is the chosen elastographic imaging technique. The implementation discussed here is clinically sound, based on a spectrally engineered illumination source that enables imaging of biological surface markers (blood vessels) with high contrast. Mechanical loading and deformation of the sample is performed using a rolling indenter, which enables the investigation of large organs (size of kidney) with reduced measurement time compared to a scanning approach. Furthermore, the rolling indentation results in strain contrast improvement and an increase in detection accuracy. The successful application of digital image correlation is first demonstrated on a silicone phantom and later on biological samples. Elasticity parameters and their corresponding four-dimensional distribution are generated via solving the inverse problem (only two-dimensional displacement field and strain map experimentally available) using a well-matched hyperelastic finite element model.

## 1 Introduction

Minimally invasive surgery has revolutionized surgical intervention. It enables a quicker recovery for the patient related to reduced postoperatory stress. Moreover, it offers some aesthetic advantages in particular for exposed parts of the human body, e.g., the face. However, these advantages come at the expense of losing the sense of touch, as palpation is an important tool for tumor localization in open surgery. The localization process is based on the increased stiffness of tumorous tissue compared to healthy tissue. Preoperational data acquired from computer tomography (CT), magnetic resonance tomography (MRT), and ultrasound imaging can reveal information about the size of the tumor and location of the transition between malignant and healthy regions within the organ, but do not account for any changes in the location or shape of the organ due to increased deformability of soft tissue in comparison to hard tissue, different hydration levels of the body compared to the time when the preoperatory data were collected or changed position of the body (e.g., supine versus lateral recumbent). Furthermore, the application of pressure, which is needed for expanding a body cavity (e.g., thorax) in order to provide the surgeon with more space for carrying out the operation, results in a deformed shape and location of the organ under investigation. The goal of the minimally invasive intervention is to remove the tumor under best possible preservation of important functional tissue such as blood vessels and nerves.

Hence, there exists an unmet need for the development of sensor systems, which supports the surgeon during minimally invasive surgery in tumor localization.

Elastography is an imaging technique that enables the recovery of quantitative elastic parameters such as strain, stress, elastic modulus (linear elastic), or shear modulus (nonlinear elastic) from displacement measurements. Elastography has proven to be a successful complementary technique with increased sensitivity and specificity for cancer detection. This is based on the fact that tumorous tissue is 7 to 14 times stiffer in comparison to healthy tissue, according to Ref. 1. An increase of specificity from 61% to 79% was reported in Ref. 2 for the combination of elastography with ultrasound imaging. Specificity expresses the ratio of true negatives in comparison to the sum of true negatives and false positives.

The goal of this paper therefore is the development of an elastographic imaging technique in combination with finite element (FE) modeling to recover quantitative elastic parameters of tissue, which can be applied in minimally invasive surgery to support the surgeon during the three-dimensional (3-D) localization and discrimination process.

The paper is structured in the following manner, after presenting the background, the methodology and measurement principle will be explained in more detail. The FE model used will be presented, followed by the experimental setup based on rolling indentation and the results achieved for the investigation of the silicone phantom. The experimentally obtained and simulated two-dimensional (2-D) displacement fields and strain maps are compared to demonstrate that a realistic FE model has been generated, which can be used to solve the direct problem and hence the recovery of elastic parameters, which can directly not be measured. It will be shown how the strain contrast can be improved and strain ambiguities be reduced when taking into account many different strain maps, which are generated in the rolling indentation process. Afterwards, the 3-D distribution for different stress tensor elements and locations of the indenter will be represented. Moreover, the successful application of the image correlation technique to a biological sample will be shown.

## 2 Background

The working principle of elastography relies on the comparison of at least two different states of the object under investigation (mechanically loaded and unloaded). The loaded state can be obtained when applying an external force or body internal forces, which are mostly associated with the cardiovascular system, such as blood pressure change.^3^ The latter is limited to the investigation of blood vessels or tissue in close proximity only. Under load, tumorous tissue deforms less than healthy soft tissue due to its increased stiffness. By comparison of the two data sets, loaded and unloaded, the elastic properties of the tissue can be obtained and tissue abnormalities be localized. In that manner small tumors and/or enlarged lymph nodes hidden underneath a layer of tissue such as peritoneum, which may not have been registered in the preoperatory data, can be found, resulting in an increased accuracy of the diagnosis. Elastography has successfully been combined with some of the most established imaging modalities such as ultrasound imaging,^2^ OCT imaging,^4^ MRI,^5^ and CT.^6^ Digital image correlation for the dynamic analysis of biomechanical systems such as bones, tendons, and muscles was implemented in Ref. 7. The application of an endoscopic electron speckle pattern interferometry) system for deformation measurement of porcine kidney is described in Ref. 8. Digital holographic distal endoscopy for 3-D shape and deformation measurement has been reported in Ref. 9. Recently, the application of photoacoustic tomography for tissue investigation and discrimination was demonstrated in Ref. 10. In Ref. 11, it could be demonstrated that recording speed and optical resolution can be increased when employing digital double exposure holography for the investigation of the deformations caused by acoustic waves. In Refs. 12 and 13, digital image correlation elastography has been used.

In addition to the different imaging modes used at different resolution levels (ultrasound, CT, MRT, and visible light-based optical techniques), elastography is not only restricted to macroscopic organ and tissue level but can furthermore be applied at a microscopic cell level. A recent technique for the determination of elastic behavior of cells is Brillouin microscopy, which is based on inelastic scattering similar to Raman spectroscopy. In this case, the application of an external force results in a spectral shift. The amount of spectral shift can be related to stiffness properties.^14^^,^^15^

Furthermore, atomic force microscopy has been applied for the investigation of individual cells, whereas the tip of the scanning diamond needle penetrates the specimen vertically while recording the force and displacement.^16^ It is worth noticing that, in contrast to macroscopic properties of tissues and organs, at microscopic level cancerous cells have a smaller Young’s modulus than healthy cells.^17^^,^^18^ The different elastic behavior of tissue and organs can be explained by a more densely meshed extracellular matrix of cancerous tissue in comparison to healthy tissue, resulting in an increased stiffness.^19^^,^^20^ The reduced stiffness of cancerous cells on the other hand may facilitate migration and invasion of cancerous cells in comparison to healthy cells. The increased stiffness of the extracellular matrix likewise promotes cell migration due to a feedback between forces.

In elastography, different types of loading can be characterized, according to Ref. 21, as quasistatic, harmonic, and transient. The quasistatic loading is the most widely used and has been applied initially in ultrasound elastography and was later extended to other elastographic imaging modalities. Harmonic loading consists of a low frequency acoustic wave transmitted within the tissue using a sinusoidal vibration source in contact mode. The local shear modulus can be obtained directly from the velocity of the propagating shear wave and the density of the specimen, as discussed in Ref. 22 for homogeneous tissue. However, shear waves attenuate rapidly in biological soft tissue, which limits the penetration depths. The problem can be overcome using transient loading, which is based on an acoustic radiation force that is locally focused within the specimen. The loading of the transient source is performed in a noncontact manner.

## 3 Methodology

The requirements imposed on the elastographic measurement system are recovery of cancer relevant elastic parameters, clinically applicable (no harm to patient), an endoscope implementable measurement principle, acquisition of data in real time, optical performance parameters as encountered in endoscopy (field-of-view typically 50×50  mm and sub-mm optical resolution^23^) and robustness against disturbing environmental conditions (air flow and vibrations). Digital image correlation elastography satisfies all these requirements. Furthermore, it can be implemented using a visible light camera, which not only reduces setup costs but also enables the extraction of other morphological features relevant to cancer, such as hypervascularization.^24^ The hypervascularization effect is characterized by an increased density of blood vessels with a randomly distributed structure and irregular branching.^24^

### 3.1 Digital Image Correlation

Digital image correlation is based on the comparison of two images at different states (loaded and unloaded). It is a well-known technique, widely applied in digital particle imaging velocimetry,^25^ in order to study fluid flow^26^ and air flow.^27^ More recently it has also been applied in biomedical imaging in order to obtain displacement vector fields.^28^^,^^29^

**Fig. 1:**
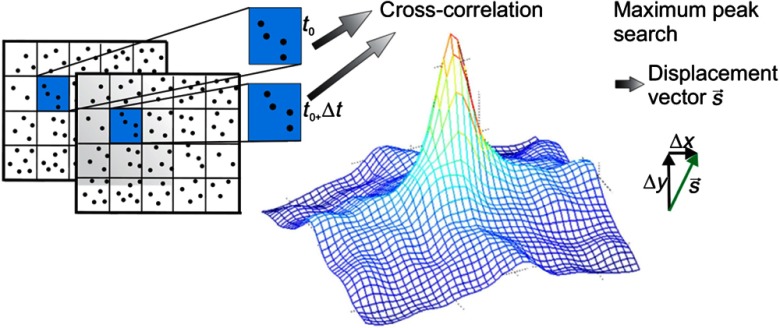
Schematic representation of the working principle for digital image correlation.

Each image is divided in overlapping subimages, also known as interrogation areas. A displacement vector is obtained by comparing the corresponding subimages of the two states using crosscorrelation, as shown in Fig. 1. Extraction of the displacement vector via crosscorrelation can computationally best be accomplished via calculation of a 2-D FFT of both corresponding interrogation areas, computation of cross product of the corresponding FFTs of the first interrogation area, the resulting conjugate of the second interrogation area, and determination of mean displacement vector from location of maximum with respect to the center.

Design rules that have to be taken into account for digital image correlation are:

– The corresponding size of the interrogation area should be larger than the diameter of the markers that are tracked (e.g., speckles and particles).
– Adjacent interrogation areas need to overlap in order to ensure that marker points that are positioned close to the edge of the interrogation area can still be tracked.
– Homogeneously distributed fine structural details with a density of at least seven marker points per interrogation area need to be available in order to obtain a detection probability of 95%.^30^
– The in-plane motion difference between individual marker points should be smaller than the diameter of a marker point.^30^
– The out of plane motion of individual marker points should be smaller than quarter the depth of field.^30^
– The maximum in-plane displacement of less than a quarter of the interrogation area employed.^30^
– The maximum in-plane rotation should not exceed 10 deg.^31^
– The maximum marker point displacement of half the size of a marker point during the camera integration time.^32^

The spatial resolution in digital image correlation matches the pixel size but can be improved successively, via the use of interpolation routines, to 0.001 pixels, as discussed in Ref. 28. The spatial separation of displacement vectors is determined by the size of the interrogation area and the overlap between consecutive interrogation areas, e.g., a 20×20  pixel interrogation area with 50% overlap results in a density of 10 pixels (for every 10 pixels a new displacement vector is calculated). From the displacement, the strain that represents a quantitative elasticity parameter can be calculated. The strain is a tensor that is defined as the partial derivative of the displacement.

$$\epsilon =\left[\begin{array}{ccc} \epsilon_{xx} & \epsilon_{xy} & \epsilon_{xz}\\ \epsilon_{yx} & \epsilon_{yy} & \epsilon_{yz}\\ \epsilon_{zx} & \epsilon_{zy} & \epsilon_{zz} \end{array}\right]=[\begin{array}{ccc} \frac{\partial u_{x}}{\partial x} & \frac{1}{2}(\frac{\partial u_{x}}{\partial y}+\frac{\partial u_{y}}{\partial x}) & \frac{1}{2}(\frac{\partial u_{x}}{\partial z}+\frac{\partial u_{z}}{\partial x})\\ \frac{1}{2}(\frac{\partial u_{y}}{\partial x}+\frac{\partial u_{x}}{\partial y}) & \frac{\partial u_{y}}{\partial y} & \frac{1}{2}(\frac{\partial u_{y}}{\partial z}+\frac{\partial u_{z}}{\partial y})\\ \frac{1}{2}(\frac{\partial u_{z}}{\partial x}+\frac{\partial u_{x}}{\partial z}) & \frac{1}{2}(\frac{\partial u_{z}}{\partial y}+\frac{\partial u_{y}}{\partial z}) & \frac{\partial u_{z}}{\partial z} \end{array}].$$ (1)

From Eq. (1), it follows that $\epsilon_{xz}=\epsilon_{zx}$, $\epsilon_{yz}=\epsilon_{zy}$, and $\epsilon_{yx}=\epsilon_{xy}$. Hence the strain tensor is a symmetric second-order tensor that can be reduced to six elements and can be represented using the Voigt notation as discussed in Ref. 33. The strain can be approximated via the displacement change that occurred for each unit length of the specimen under investigation.^34^ The unit length is usually represented by the pixel-size in the object space. In our case, two parameters of the strain tensor or strain vector can be calculated, which can be derived from the displacements in the x and y directions. In our case, the indenter is actuated in the y direction; therefore, the largest displacement occurred along this direction. Therefore, the corresponding elastic parameters along the y direction will predominantly be considered in the following.

### 3.2 Inverse Problem and Three-Dimensional Finite Element Modeling-Based Solution

**Fig. 2:**
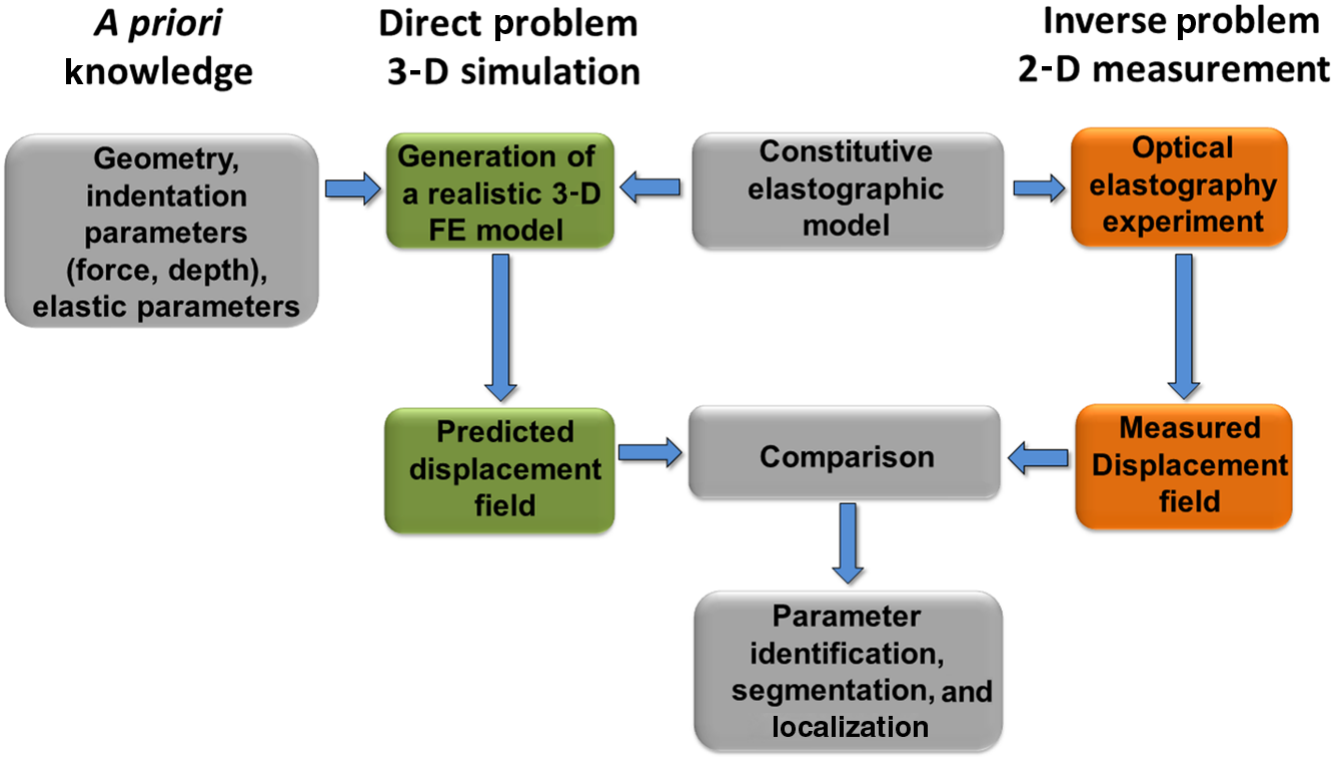
Parameter-identification based on the comparison between the outcome of the FE-model (solution of direct problem) and experimentally measured values (displacement field and strain map).

In addition to the 2-D strain map, which can be recovered from the displacement field, other elasticity parameters can be obtained only at the interface between indenter and specimen. Knowledge of the indenter geometry, the force applied, and the displacement obtained at the indenter position enables the recovery of elastic parameters such as stress and shear modulus for only this point. However, the goal is to retrieve the elastic parameters for all imaged object points and to show their corresponding 3-D distribution. The 3-D information is essential in order to locate the tumor in minimally invasive surgery and hence to support the navigation process. Hence, a solution to an under-defined system needs to be found in order to recover the spatial distribution of elastic parameters. An inverse problem has to be solved, which can be accomplished via solving a direct problem. The underlying physical laws applicable to both, inverse and direct problem, are the same, but inverted, and relate the elastic properties to a measurable mechanical response. The direct problem is well defined with respect to the geometrical (dimensions of tissue and foreign body, indenter geometry, and force) and elastic input parameters (elastic modulus for linear elastic, shear modulus, and locking stretch for nonlinear elastic). It starts with the desired elastic parameters as input parameters, whereas the experimentally obtained parameters (displacement field and strain map) represent the output, which can be compared with the measured values, see Fig. 2. Hence, it is an inverted process of the inverse problem and is therefore referred to as a forward problem.^21^ In that manner, the desired unknown elastic parameters, which experimentally cannot be retrieved, can be obtained from a realistically well-matched FE model that enables generating the computational solution of the direct problem. Solving the direct problem can be implemented using a noniterative and iterative approach. The noniterative approach requires a good estimate of the input parameters that can be generated from *a priori* knowledge based on stress–strain measurements about the different materials/tissues and the geometry of the indenter and object under investigation. The generation of relevant data to apply the noniterative method for medical applications can be obtained from previous operations on patients of similar conditions (age, gender, and life style) or preoperational data. Moreover, the geometry/morphology of inner organs such as kidney and the locations of abnormalities (cancer) can be obtained from preoperational data such as CT and MRT data.

The noniterative approach enables a quicker recovery of elastic parameters in combination with computational methods such as the FE method. The iterative approach on the other hand requires less input parameters; usually only the displacement field and the general elastic behavior (nonlinear hyperelastic behavior is very common among soft tissue^35^) of the specimen are provided. The loading and elastic parameters are iteratively adjusted to result in a good match for the modeled and measured displacement field. Under the assumption that the same constitutive model is applied, the iterative approach is more time consuming, e.g., 6000 iterations have been employed in Ref. 36 to obtain a good match.

Many FE models of different dimensionality and elastic properties have been used for the implementation of the direct problem, among others a 2-D hyperelastic Arruda Boyce FE model,^1^ a 3-D axisymmetric FE model of linear elastic behavior,^34^ and a four-dimensional (4-D) hyperelastic model taking into account the temporal component.^37^ A detailed discussion of the many different existing models used in elastography to implement the direct problem can be found in Ref. 21. Since our measurement principle is based on rolling indentation, a 4-D hyperelastic FE model has been generated, taking into account the three spatial dimensions and the changing location of the indenter over time. The chosen constitutive FE model to simulate the hyperelastic behavior of soft tissues is the Arruda–Boyce model.^38^ Besides the excellent modeling of the nonlinear elasticity behavior of soft tissue, it furthermore offers the advantage of requiring only two parameters, shear modulus G and locking stretch $\lambda_{m}$. The shear modulus is defined as the ratio between shear stress and shear strain whereas the locking stretch corresponds to the value of the chain stretch at which the chain length reaches its fully extended state. A more detailed discussion can be found in Ref. 38.

$$\sigma_{0}=G[(\epsilon^{2}-\epsilon^{-1})+\frac{1}{5\lambda_{m}^{2}}(\epsilon^{4}-\epsilon^{-2})+\frac{11}{175\lambda_{m}^{4}}(\epsilon^{6}-\epsilon^{-3})+\frac{19}{875\lambda_{m}^{6}}(\epsilon^{8}-\epsilon^{-4})+\frac{519}{67,375\lambda_{m}^{8}}(\epsilon^{10}-\epsilon^{-5})].$$ (2)

The parameters $\sigma_{0}$ and ϵ in Eq. (2) represent stress and strain, respectively.

### 3.3 Silicone Phantoms and Corresponding FE Models

**Fig. 3:**
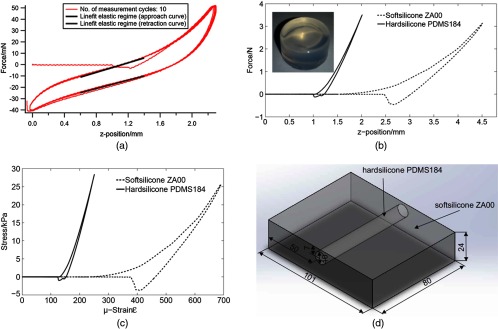
Uniaxial compression test results: (a) force indentation diagram for porcine kidney highlighting its hyperelastic and viscoelastic behavior, (b) force-indentation diagram obtained from cylindrical silicone samples of hardsilicone and softsilicone (dimensions in mm), (c) corresponding stress–strain diagram, and (d) dimensions of silicone phantom.

As a proof of concept, before examining the morphologically highly complex kidney, a silicone sample of similar dimension and hyperelastic behavior has been created, which is common practice in optical elastography.^1^^,^^36^^,^^34^ In order to generate a realistic silicone phantom, the elastic behavior of porcine kidney has first been studied in an uniaxial compression test, carried out on a commercial microindenter Zwicki line 500 (Roell GmbH, Ulm, Germany). The hyperelastic behavior mentioned in Ref. 35 could be confirmed, see Fig. 3(a). Afterward, various materials such as, Agar-Agar and different types of silicone ZA-OF, ZA-OO (O ShA) (ordered from Polymerschmiede GmbH), and PDMS Dow Corning 184 (ordered from Biesterfeld Spezialchemie GmbH) have been investigated to mimic the biological tissue. It was found that ZA-OO (O ShA) was best suited for mimicking the healthy soft tissue and PDMS Dow Corning 184 for mimicking the pathological tissue. By comparison with the force indentation curve for a porcine kidney shown in Fig. 3(a), a similar hyperelastic behavior and viscoelastic behavior of the silicone sample, shown in Figs. 3(b) and 3(c) could be confirmed. Moreover, the silicone samples also enable good reproducibility and temporal stability (not the case with Agar-Agar). The stress–strain curves obtained, as shown in Fig. 3(c), could be used as *a priori* information in order to generate a realistic FE model.

**Fig. 4:**
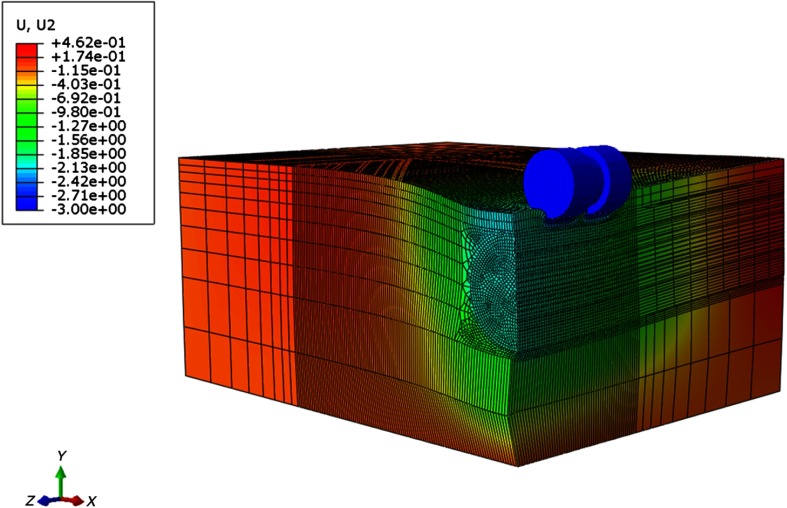
3-D FE model showing the displacement along y-direction of silicone phantom and indenter, sliced at central location of foreign body (parameters applied 3 mm indentation, indenter located on top of foreign body).

In Fig. 3(d), the dimensions of the phantom with a foreign body are shown. In correspondence to the phantom, an FE-model with a centrally located cylindrical inclusion of increased shear modulus (10 mm diameter) has been generated. The same outer dimensions have been used to produce another homogeneous silicone phantom to model an entirely healthy organ, which will be used to improve the strain contrast, which will be discussed in more detail at a later stage in this paper. The coordinates of indentation with respect to the edge of the phantom (8 mm), and the geometry of the indenter (two miniature ball bearings with 6 mm diameter and 3 mm width), which introduces the loading, have been taken into account for the generation of a realistic 3-D FE model. The entire FE model is composed of 110,565 elements. The size of the FEs ranged from $0.3\times 0.3\times 0.3\;{\mathrm{mm}}^{3}$ to $5\times 5\times 5\;{\mathrm{mm}}^{3}$. Small mesh sizes were employed in contact areas, i.e., in the contact area between the indenter and the tissue model and in the contact area between the homogeneous tissue and the foreign body (see Fig. 4).

In order to obtain a realistic modeling of the elastic behavior of malignant and normal tissues, different combination ratios between hard (PDMS 184) and soft-silicone (ZA-OO) have been generated and the force-indentation curves have been obtained from uniaxial compression tests applied to cylindrical silicone samples, as shown in Fig. 3(b). The stress–strain curves for soft and hard silicone were used as input parameters for the FE model. A 100% soft-silicone (ZA-OO) was chosen for mimicking normal tissue and a 100% hard-silicone (PDMS Dow Corning 184) for malignant tissue. The shear modulus G and locking stretch $\lambda_{m}$ for soft silicone (G=0.73  kPa and $\lambda_{m}=7.7$) and hard silicone (G=7.73  kPa and $\lambda_{m}=7$) obtained from the stress–strain curve [generated by uniaxial compression test, see Fig. 3(b)] have been used as input parameters for the FE model. These values are within the naturally occurring range according to Ref. 1 (malign tissues are 7 to 14 times stiffer in comparison with normal tissue).

Figure 4 shows the 3-D displacement field for a centrally located indenter. It took 2.5 h to compute the FE model, which was run on the bwUniCluster (one node and seven processors).

## 4 Experimental Results

### 4.1 Elastographic Measurements

**Fig. 5:**
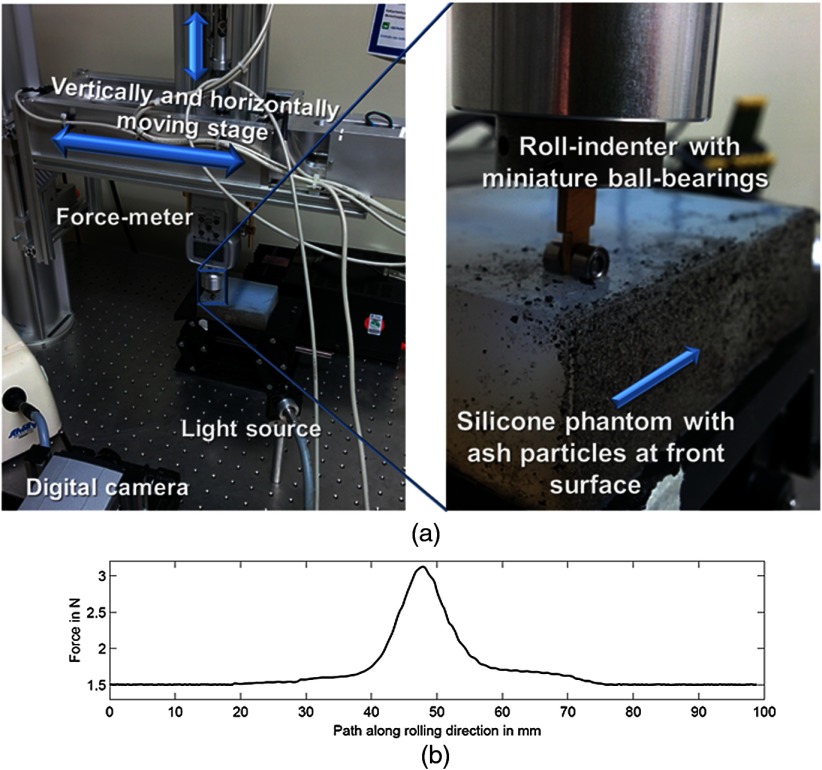
(a) Setup: roll-indenter with silicone phantoms, (b) graphical representation of the force applied along the indenter’s rolling direction with and without foreign body.

In order to apply a well-known force and indentation over the entire length of the organ within a minimum amount of time, a two-axis force rolling indenter was manufactured in the institute engineering workshop, see Fig. 5(a).

The rolling movement is well suited for minimal invasive surgery, based on the fact that large areas can be scanned within a short time, in comparison to a pointwise measurement mode. Rolling indentation is related to the “quasi static loading,” as described in Ref. 21, albeit with temporally changing local coordinates of the indenter.

The force rolling indenter supports two measurement modes, namely constant indentation and constant force. Both are made possible via a feedback loop between the force-meter (Alluris FMI-B30BI, measurement range: 0 to 10 N, measurement uncertainty: 15 mN), and the stepper motor driving the z-axis (range: 19 mm, positioning uncertainty: 10  μm). The rolling indenter can be displaced laterally by 156 mm with a positioning uncertainty of 50  μm.

Furthermore, the device enables quick change of indenters with different geometries and sizes.

The aim is to use a small indenter in order to reduce the influence of disturbing artifacts, such as inhomogeneities that lie outside the imaged area and of an uneven measurement surface, on the measurement result.^39^ However, the size of the indenter cannot be too small since it may otherwise pierce into the sample instead of rolling across it. Moreover, in order to carry out the digital image correlation technique, the indenter size needs to match the density of the structural details available at the specimen. An indenter too small introduces strongly localized deformation, which at a small density of structural details, may not be detectable via digital image correlation, as pointed out in the design rules for digital image correlation in Sec. 3.1 based on Ref. 30 (seven markers per interrogation area).

At the next stage, a silicone phantom with a cylindrical inhomogeneity mimicking malign tissue, in accordance with the FE model has been generated. For mimicking normal tissue 100% soft-silicone (ZA-OO) was chosen and for malign tissue 100% hard-silicone (PDMS Dow Corning 184). The front surface of the silicone phantoms was covered with markers consisting of ash particles with different sizes (300  μm to 1.3 mm) and shades (light gray to black), to enable a dense distribution of tracers while ensuring a good discrimination between adjacent markers. A strainer was used to restrict the particle size to 1.3 mm. Prior to the application of the ash particles the silicon phantom has been cleaned by water, resulting in a stronger adhesion of the ash particles. The ash particles have then been dispensed on the phantom. Afterward the front surface of the phantom was oriented toward the floor and shacked several times to remove those particles, which are not well fixed on the surface of silicon phantom. These particles would otherwise during the rolling indentation process fall off and hence result in measurement errors.

The silicone sample was illuminated with a white light source. Two miniature ball bearings with 6 mm diameter and 3 mm width have been used for the indenter, as shown in Fig. 5(a) enlarged image on right-hand side. The indentation depth employed was 3 mm. The indenter was placed at a distance of 8 mm to the front surface of the silicone phantom. The rolling indenter was moved at a speed of 2.4  mm/s across the specimen. A digital camera (Zyla 5.5, 2560×2160  pixels, 6.5  μm pixel size) in combination with a Tamron 08831 camera objective was used to record the images. The resolution of the system is in our case not determined by the camera objective, which can deliver an NA of 0.03 (20  μm resolution) at 200 mm recording distance, but by the magnification of the camera objective of 0.115 and the sensor’s pixel-size, resulting in resolvable features of 56  μm. In that manner renal blood vessels (interlobular arteries and veins) with perimeter size ranging from 150  μm to 200  μm^40^ can well be resolved and imaged. The field of view recorded by the camera was 144  mm×121  mm.

In order to save memory and more importantly postprocessing time, the images have been cropped to 1800×520  pixels, which corresponds to a field of view of 101  mm×29  mm. Two-hundred images have been recorded with an exposure time of 0.04 s (25 fps). This exposure time was well chosen in order to ensure that motion artifacts of the rolling indenter do not influence the measurement. According to Ref. 32, the lateral movement in the image plane is limited to half the size of the smallest imaging detail. In our case, the rolling indenter moved roughly 100  μm during the exposure time, which is perfectly suitable for the particles employed in our experiments. Hence, two different object states can be recorded within a time frame of less than 100  μs.

In addition to the task of applying a load, the indenter can also be used to measure the force along the direction of indentation, as shown in Fig. 5(b). In this manner, the indenter fulfills the functionality of an elastographic sensor, which in combination with the 2-D displacement field and its derived elastic parameters, can be used for 3-D location of the elastic inhomogeneities.

**Fig. 6:**
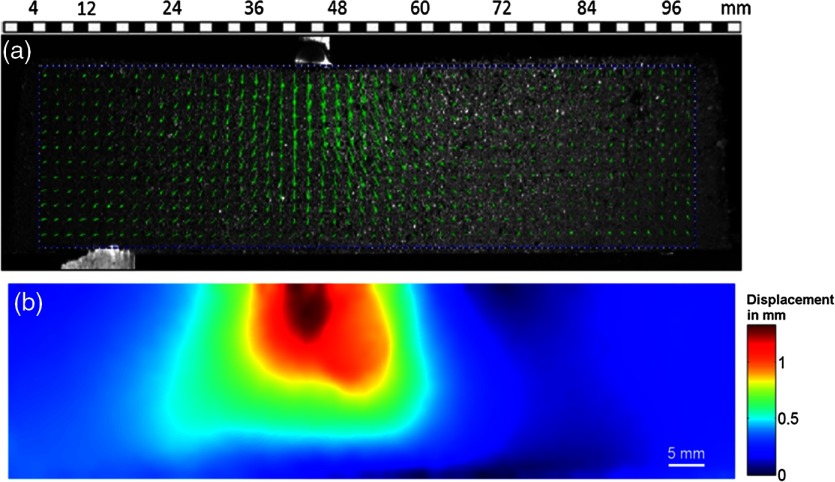
Experimental results: displacement maps obtained: (a) displacement vector field and (b) contour map representation (absolute value of displacement vector).

The recorded images were analyzed using PIVLab.^41^ An interrogation area of 65×65  pixels was used with an overlap of 50%. Hence, displaced marker points that are positioned at the edge of the interrogation window could be tracked. The interrogation area was 3.6 mm while the largest marker size was 1.3 mm, which means that more than seven marker points fall within an interrogation area. In a preprocessing step, the contrast of the images was increased using histogram equalization, by which detection probability for valid vectors in images could be increased fivefold.^42^ Moreover, it was possible to increase the spatial resolution of the displacement vectors by iteratively changing the size of the interrogation window, whereby an increase of resolution down to 0.04 pixels was reported in Ref. 43. Moreover, a 2×3 Gaussian interpolation was performed on the cross-correlation peak, which reportedly increases the resolution, as discussed in Ref. 44. Combination of both the effects results in a resolution for the displacement vectors of 0.02 pixels. The displacement vector field and its contour map are displayed in Fig. 6.

### 4.2 Comparison with FE-Model

**Fig. 7:**
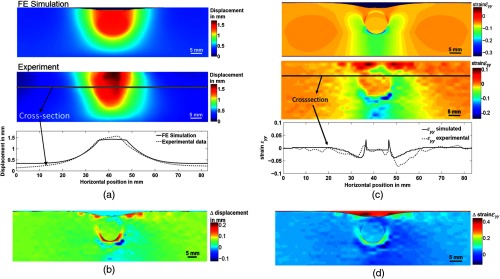
Comparison between FE simulation and experimentally obtained results (top FE Simulation, center experimentally obtained displacement field, cross-section plot, highlighted by dark line in experimental data) (a) displacement field, (b) strain map, (c) difference map between simulated and measured displacement field, and (d) difference map for simulated and calculated strain.

Subsequently, the displacement fields and cross-section plots of the FE simulated data were compared with the experimental data obtained for the silicone phantom with a foreign body, see Fig. 7(a). The difference map between simulated and experimentally obtained displacement fields is displayed in Fig. 7(b). A good overall match was obtained with a mean measurement error of 0.0604 mm and a standard deviation of 0.0654 mm, which have both been calculated from the difference map displayed in Fig. 7(b).

High-displacement values are preserved when the pressure wave passes through the foreign body due to their increased stiffness in comparison to the soft silicone, which already indicates the location of the foreign body. However, the displacement does not represent an elastic parameter.

Therefore, the strain $\epsilon_{yy}$ along the direction of indentation (y-direction) has been calculated, which in addition to representing an elastic parameter, enables the visualization of elastic inhomogeneities with high contrast. In addition to the high positive strain values of the experimental strain map located at the upper edge of the silicone phantom, a similar strain distribution between the FE model and the experimentally obtained strain map was observed; see Fig. 7(c). The mean error for strain difference displayed in Fig. 7(d) was 0.0598 with a standard deviation of 0.0562. A way to overcome the identification ambiguity arising from the high strain values at the edge of the phantom will be discussed in Sec. 4.3 based on strain map averaging.

In conclusion, a good agreement between FE modeling and experiments could be achieved for the displacement and strain along the indentation direction.

**Fig. 8:**
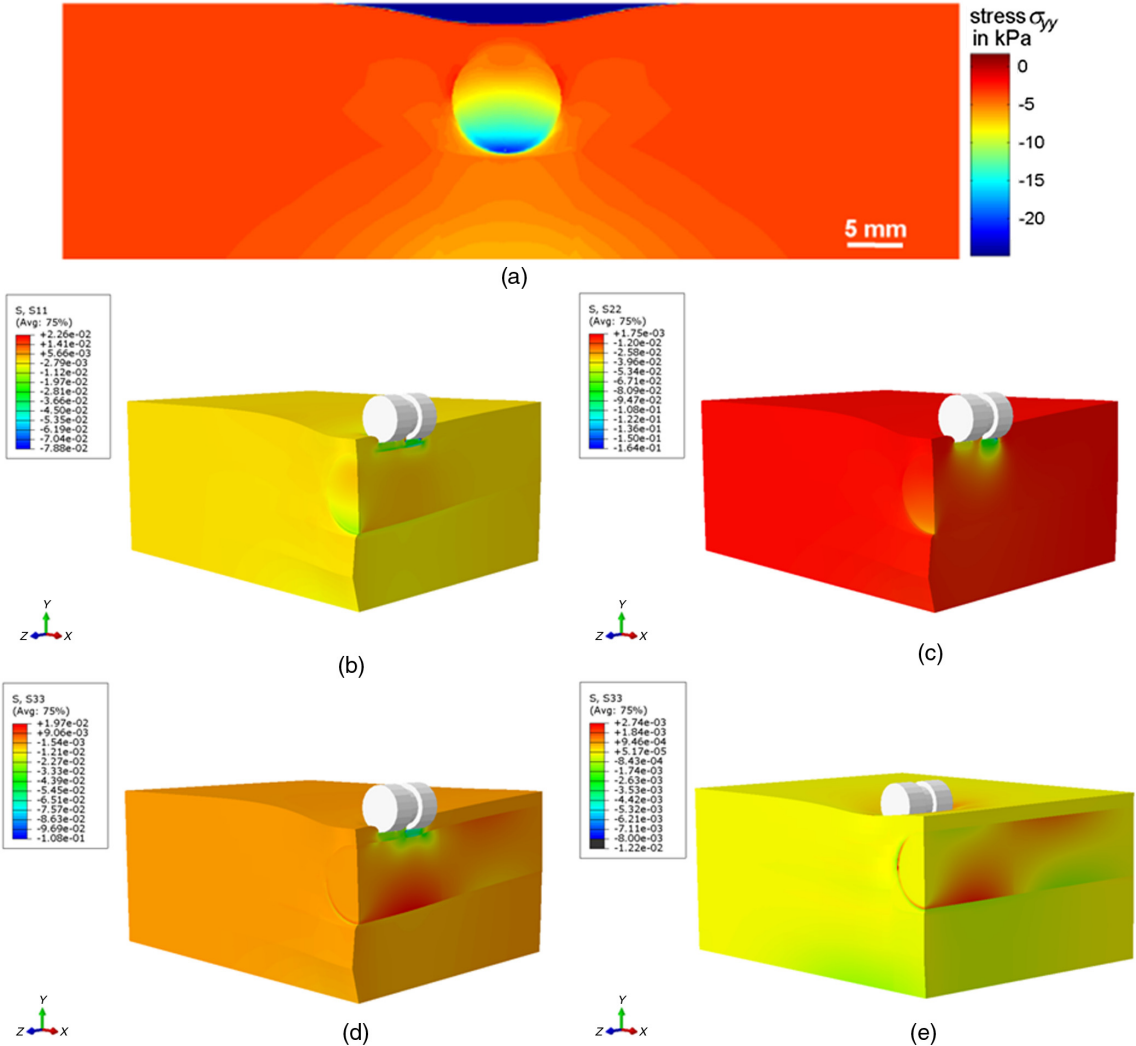
Stress distributions obtained using the hyperelastic Arruda–Boyce model for Abaqus (a) $\sigma_{xx}$ at the front surface, (b, c, d) 3-D stress distribution along (b) the x-direction $\sigma_{xx}$, (c) y-direction $\sigma_{yy}$, (d) and z-direction $\sigma_{zz}$ with indenter positioned on top of inhomogeneity, (e) 3-D stress distribution along z-direction $\sigma_{zz}$ with indenter positioned in 10 mm distance to inhomogeneity.

Therefore, the FE model represents a realistic model that can be employed to reveal other parameters, which can only be obtained solving the inverse problem. This, in particular, refers to the stress distribution. FE modeling enables the recovery of the strain and stress tensors in 3-D, as shown in Figs. 8(a)–8(d) for $\sigma_{xx}$, $\sigma_{yy}$, and $\sigma_{zz}$. Moreover, the stress tensor at different indenter positions can be recovered. To illustrate the changed indenter location, the 3-D stress distributions $\sigma_{zz}$ obtained from FE modelling are displayed in Fig. 8(d) for the indenter location right above the foreign body, and Fig. 8(e) for the indenter location at 10 mm distance from the foreign body.

### 4.3 Strain Contrast Enhancement Techniques

Two different techniques to enhance the strain map will be discussed. The first is based on the additional recording of displacements fields from an elastically homogeneous specimen. The second approach is based on the averaging of multiple strain maps, which have become available due to the application of the rolling indenter.

**Fig. 9:**
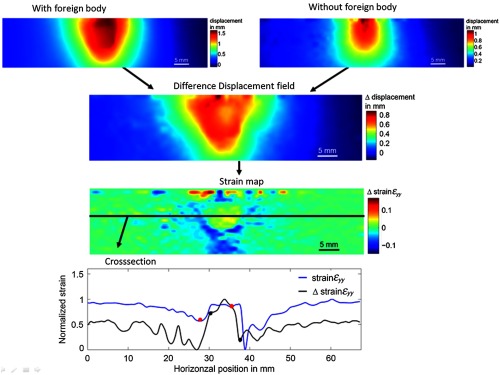
Flow chart and results for obtained displacements fields with and without foreign body, the resulting difference displacement field, the calculate strain field, and corresponding cross-section plot, which compares the difference approach strain field with the conventional results shown in Fig. 7(b).

The first approach was implemented subtracting the displacement field obtained from a silicone phantom from the one obtained from the silicone phantom with a foreign body. The position of the foreign body can already be seen in the difference displacement map, see Fig. 9. Finally, a difference strain map $\Delta \epsilon_{yy}$ was calculated from the difference displacement map and an improved contrast could be achieved, as highlighted in the cross-section plot in Fig. 9. For calculating the strain contrast, the smallest maximum and highest minimum have been taken into account and the strain cross-sections normalized for better comparison. The significant values for the normal strain map [shown in Fig. 7(c)] are indicated by the red dots, whereas black dots have been used for indicating the difference strain map, see Fig. 9. A contrast of 0.20 was obtained for the normal strain map, whereas an improvement to a contrast value of 0.64 for the difference strain map could be obtained.

**Fig. 10:**
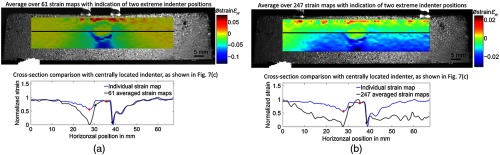
Averaging the strain maps obtained from: (a) 61 recorded indenter position, (b) 247 different indenter positions, which demonstrates that the high strain values at top edge of the strain map arise from the indenter. The cross-section plot at the bottom of each subfigure highlights the contrast improvement for identifying the foreign body in comparison to the individual strain map obtained from the centrally position indenter shown in Fig. 7(c).

The second approach is based on averaging, which is made possible by the large amount of displacement fields and consequently strain maps produced by rolling indentation. This approach does not only enable the improvement of strain contrast in order to support the identification of the foreign body but also minimizes the detection ambiguity caused by high strain values in close proximity to the indenter position. In Fig. 10, the averaging results obtained from two different amounts of strain maps [61 strain maps in Fig. 10(a) and 247 strain maps in Fig. 10(b)] are shown. It becomes obvious that the positive high strain values at the top edge of the strain map are caused by the indenter, which when moved across the specimen results in an upward directed displacement within its close proximity. These artifacts, which for a single strain map lead to a false interpretation [see Fig. 7(c)], can therefore be associated to the indenter. They can hence be neglected for the identification of the foreign body. To evaluate the detection improvement, the contrast within close proximity of the foreign body has been calculated. For calculating the contrast, the smallest maximum and highest minimum have been taken into account, as indicated by the red (individual) and black (averaged) dots in Fig. 10. For the individual strain map a contrast of 0.20, for averaging 61 strain maps a contrast of 0.92 and for averaging 247 strain maps a contrast of 0.71 could be obtained. Hence, the contrast improvement is restricted to rolling indentation, which is located in close proximity of the foreign body. The averaging approach highlights the advantage of the rolling indentation approach, which enables a strain contrast improvement after the data have been selected. It can furthermore be fine-tuned with respect to the chosen amount of data to result in the best possible contrast.

The implementation in an FEM environment for the difference strain map method should not be too problematic, whereas the averaging method will be more challenging due to the large amount of data that will have to be created.

### 4.4 Porcine Kidney

Conventionally, digital image correlation on biological objects is implemented with incoherent light, such as white light, while the surface of the object under investigation is spray coated in order to generate surface-localized markers that can be used to calculate the displacement field.^29^^,^^45^ Spray coating is not possible in a surgical environment. To complicate things further, no structural details are available under visible white light conditions on the porcine kidney surface. Commonly, this problem can be overcome via the application of coherent light to produce speckles. However, speckles strongly fluctuate due to the interaction of the coherent light with the biological tissue (absorption, dehydration, and multiple interference from different tissue layers). In fact, the correlation time of speckles is only a few milliseconds.^46^ However, the renal capsule hosts a network of capillaries called glomerulus, at which the blood filtration takes place. Applying a spectrally adjusted illumination, which is sensitive to strong absorption of hemoglobin, can help to visualize these capillaries, so that they can be used for digital image correlation. Therefore, the spectral properties of kidney tissue had been obtained first. For realistic measurements, fresh samples from a nearby slaughter house (Huber Fleischzentrum OHG) have been shock frozen using liquid nitrogen at 77 K in order to preserve the morphology and to prevent the appearance of ice crystals. Afterward, they were slowly warmed up to −25°C so that the tissue does not split when cutting it. A microtom (Leica CM1900) was employed for cutting the tissue in 60-μm-thick sections, which were then embedded between a microscopy slide and a cover slip. Two spectral measurements (spectrometer from MUT GmbH, 450 to 750 nm wavelength range and 0.9 nm spectral resolution), one with and one without the sample, have been taken.

**Fig. 11:**
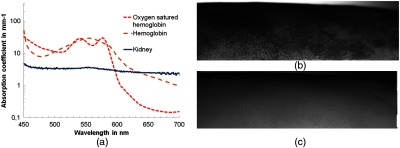
(a) Absorption coefficient hemoglobin and kidney, (b) section of kidney illuminated with LED 505-nm peak wavelength, and (c) same section of kidney illuminated with white light.

**Fig. 12:**
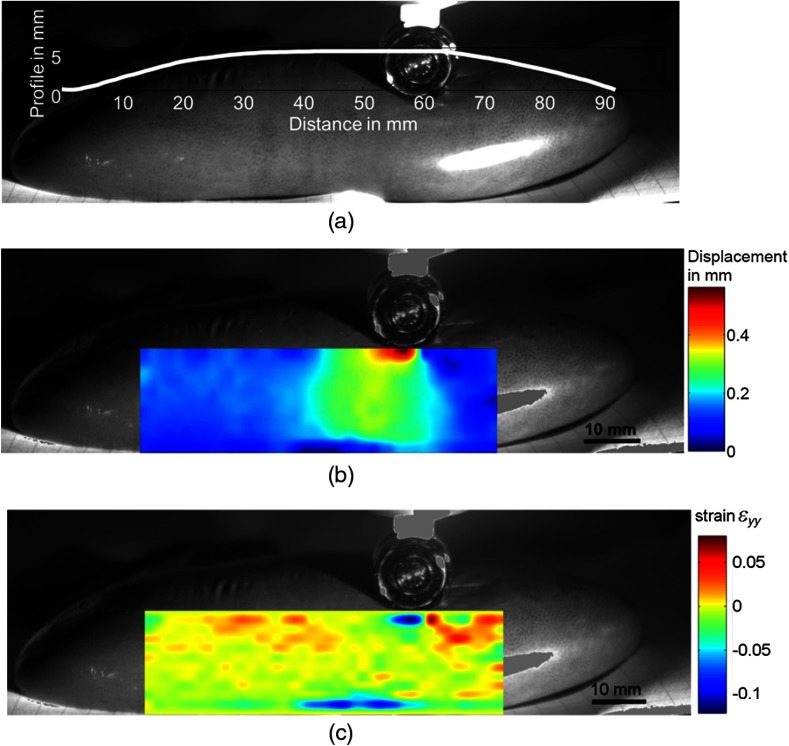
(a) Reference shape measurement using a constant force of 0.1 N, (b) displacement field obtained via digital image correlation from a porcine kidney, and (c) strain map for kidney.

The recording parameters that represent multiplicative factors, such as integration time, recording area, quantum efficiency, and electron-to–digital count conversion, have not been changed during the two measurements. Hence, a conversion from counts into radiant flux ϕ was not necessary to calculate the degree of transmission T, which enables the recovery of the absorption coefficient according to Beer–Lambert law^47^

$$T=\frac{\phi_{\text{out}}}{\phi_{\text{in}}}=\frac{{\text{Count}}_{\text{out}}}{{\text{Count}}_{\text{in}}}=e^{-\alpha \cdot d},$$ (3)

where d is the thickness of the sample, ${\text{Count}}_{\text{in}}$ is the measured spectrum without sample, ${\text{Count}}_{\text{out}}$ with sample, and α is the absorption coefficient. The absorption coefficients (log scale) of kidney (own measurements) and hemoglobin (oxygen saturated and normal, obtained from Ref. 48) are plotted in Fig. 11(a). It shows a strong absorption of hemoglobin in the blue and green spectral region in comparison to kidney. Therefore, the initial white light source used in the setup [Fig. 5(a)] was replaced by a 505-nm peak wavelength LED (Roithner C20A1-HC-30) to obtain an increased contrast of the capillaries, as comparatively demonstrated in Figs. 11(b) and 11(c). The contrast for both images was calculated using the Michelson contrast formula, while taking into account the 10 largest and smallest values to reduce the influence of image noise. An increase from 0.41 for the initial white light source to 0.61 using the green LED could be noticed. Moreover, compared to the silicone sample, the deformation was very localized and not distributed over a long distance. Therefore, the 6-mm-diameter indenter was replaced by a 10-mm-diameter one. The indentation depth applied was 2 mm. In order to ensure a constant indentation depth, a reference shape measurement with a contact force of 0.1±0.05  N had been recorded, see Fig. 12(a). The profile values have been stored and the finally applied indentation has been added to these values. The displacement field and strain map obtained are shown in Figs. 12(b) and 12(c), respectively. The indenter position and the kidney have been used for the background, whereas disturbing strong reflections at kidney and indenter have been removed in an image postprocessing step and replaced by the mean intensity of the image.

The approach of tracing the displacement map using the capillaries offers the additional benefit of enabling further tissue discrimination based on the hypervascularization effect in malignant tissue.

## 5 Discussion and Conclusion

A 4-D FE-model has been generated taking into account the 3-D spatial coordinates and the changing indenter position over time. The underlying constitutive FE-model applied is the Arruda–Boyce model, which is well suited for the nonlinear elastic behavior of soft tissue since it is hyperelastic. Furthermore, it requires only two elastic input parameters, locking stretch and shear modulus. A first guess of these parameters has been obtained from a uniaxial compression test on well-defined cylindrical silicone samples and the corresponding resulting stress–strain curves, which were then used as input parameters for the FE model. A good match for the 2-D displacement field and strain maps generated from the 3-D FE-model and the experimental 2-D digital image correlation technique using the rolling indenter could be obtained.

It could be demonstrated that a realistic FE model enables the recovery of elastic parameters of the object under investigation at different indenter positions. In this manner, the inverse problem could be solved. It was furthermore demonstrated that the strain contrast could be increased via computing the displacement difference map, based on the recording of the displacement map of a homogeneous sample and via the averaging approach, which furthermore enables the reduction of strain ambiguities that result as a disturbing artifact from the indentation process. In reality, the homogeneous sample required for the first strain contrast improvement method could be a healthy sample or region of the sample. An instrument enabling the application of well-defined experimental parameters has been developed. Future research will be focused on the miniaturization of the setup, to enable its implementation in a minimally invasive tool. A possible solution to implement rolling indentation has been demonstrated in Ref. 49, albeit without the application of digital image correlation.

The issue of missing structural details for the application of digital image correlation for the investigation of kidney could be overcome via a spectrally adjusted illumination source in the green wavelength range.

At this wavelength, a good absorption contrast of hemoglobin in comparison to kidney tissue could be obtained. Further future work will be focused on the extension to kidney sample with a foreign body, its FE modeling implementation and the measurement of 3-D displacement fields.
